# Patterns and risk factors of hyperkalemia recurrence among heart failure patients

**DOI:** 10.3389/fcvm.2026.1745946

**Published:** 2026-06-11

**Authors:** Christopher G. Rowan, Geraldine Francisco, Raymond C. Chang, K. Arnold Chan, Ravi Dhingra

**Affiliations:** 1 COHRDATA, INC, San Clemente, CA, United States; 2 US Medical, Biopharmaceuticals, AstraZeneca, Wilmington, DE, United States; 3 TriNetX, LLC, Cambridge, MA, United States; 4 Division of Cardiovascular Medicine, Froedtert and Medical College of Wisconsin, Milwaukee, WI, United States

**Keywords:** heart failure, hyperkalemia, predictors of recurrence, real world data (RWD), risk factors

## Abstract

**Background:**

Hyperkalemia, a life-threatening electrolyte condition, is linked to arrhythmias and sudden death, but evidence on its recurrence in heart failure (HF) patients is limited.

**Methods:**

We conducted a cohort study using de-identified EHR data from TriNetX Dataworks USA (study period: January 1, 2019, to June 30, 2023). HF patients who experienced hyperkalemia were included. Study outcomes included the percentage experiencing recurrent hyperkalemia (K+ >5.0 mmol/L), number and timing of recurrences, and K+ at each recurrence. Risk factors for hyperkalemia recurrence were identified using multivariable logistic regression and validated with Monte Carlo simulation. Subgroup analyses were performed for patients without baseline CKD (i.e., eGFR >60 mL/min/1.73 m² and no CKD/ESRD diagnosis).

**Results:**

The study cohort comprised 15,512 patients (median age 68 years, 42.3% female, 60.5% White, 21.4% Black). Within 12 months, recurrent hyperkalemia was commonly observed in the full cohort (49.5%) and No-CKD subgroup (37.3%). Mean number of recurrences was 2.5 and 2.0, respectively; mean K+ at each recurrence was ∼5.5 mmol/L; and time to recurrence decreased with each successive event. Significant risk factors for recurrence included history of hyperkalemia (OR 2.3, 95% CI: 2.1–2.5), hypertension, advanced CKD, immunosuppressant therapy, and malignancy.

**Conclusions:**

In this real-world cohort of HF patients, hyperkalemia recurrence was common regardless of renal function status, with increasing frequency and decreasing time intervals between subsequent recurrences. History of hyperkalemia was the strongest risk factor for recurrent hyperkalemia. Further research focused on hyperkalemia management in HF patients is warranted to prevent future recurrences and improve patient outcomes.

## Introduction

Hyperkalemia is a significant clinical concern in patients with heart failure (HF) (1). Its occurrence can precipitate life-threatening arrhythmias and increase the risk of sudden cardiac death (2–5). Population-based studies estimated that hyperkalemia occurs in 7%–40% of HF patients, with the prevalence influenced by disease severity, comorbidities, and pharmacotherapy (6–8).

Several risk factors contribute to the development of hyperkalemia among HF patients. Chronic kidney disease (CKD) and diabetes mellitus, two common comorbidities, impair renal potassium excretion, predisposing HF patients to hyperkalemia (9). Additionally, the use of renin-angiotensin-aldosterone system inhibitors (RAASi)—a cornerstone of HF management due to their mortality benefits—further increases the risk of hyperkalemia (10). Patients with advanced HF, particularly those with concomitant CKD or diabetes mellitus, are at heightened risk for hyperkalemia (7). The interplay of these comorbidities and pharmacologic therapies complicates the management of hyperkalemia in HF patients (11).

The recurrence of hyperkalemia is a critical concern, as repeated episodes can lead to the discontinuation or down-titration of essential HF therapies, potentially worsening patient outcomes (10, 12). Despite the recognized importance of this issue, research specifically investigating patterns of and risk factors for recurrent hyperkalemia in the HF population remains limited. Identifying these risk factors is essential for developing and utilizing targeted interventions to prevent hyperkalemia recurrence and optimize HF management. Hence, we designed the present study to examine hyperkalemia recurrence and to identify independent baseline risk factors for hyperkalemia recurrence in patients with HF. By elucidating these risk factors, this study aims to identify HF patients at high risk of hyperkalemia recurrence, inform clinical strategies to mitigate hyperkalemia recurrence and improve outcomes in the HF population.

## Methods

A retrospective, observational cohort study was conducted using electronic health record (EHR) data from the TriNetX Dataworks USA Network (13–16), during the study period of January 1, 2019, to June 30, 2023. The data, encompassing both outpatient and inpatient records, were sourced from 52 US-based healthcare organizations (HCOs) serving approximately 90 million patients, including academic medical centers, integrated delivery networks, specialty hospitals, and large specialty physician practices, and were generated from routine healthcare encounters within this “open network.” All data were standardized using terminologies such as the International Classification of Diseases, Tenth Revision, Clinical Modification (ICD-10-CM) for diagnoses, Current Procedural Terminology (CPT), Healthcare Common Procedure Coding System (HCPCS), and International Classification of Diseases, Tenth Revision, Procedure Coding System (ICD-10-PCS) for procedures, RxNorm for medications, and Logical Observation Identifiers Names and Codes (LOINC) for laboratory results.

**Figure 1:**
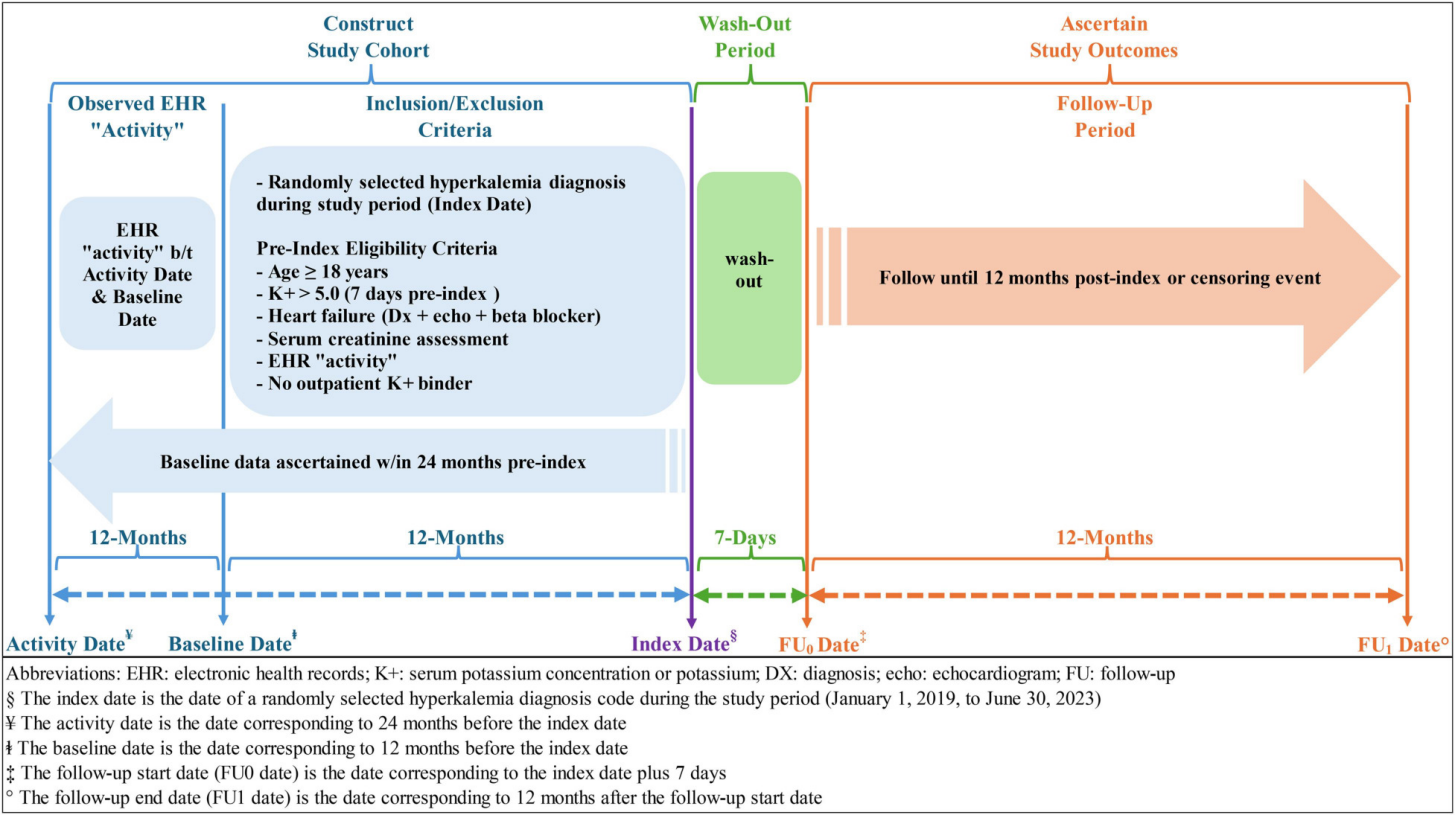
Study schema.

The study cohort included adults aged ≥ 18 years with a hyperkalemia diagnosis (randomly selected during the study period = index date) and a serum potassium (K+) concentration > 5.0 mmol/L within 7 days pre-index (Figure 1). Random selection of the hyperkalemia diagnosis aimed to capture both incident and prevalent hyperkalemia cases. Further eligibility required a baseline HF diagnosis, an echocardiogram, and a beta blocker prescription, as well as a serum creatinine measurement and at least one healthcare encounter in the EHR network. Patients were excluded from the study if they had an outpatient prescribing record for a potassium binder within the 12 months pre-index (i.e., sodium polystyrene sulfonate, patiromer, or sodium zirconium cyclosilicate).

Within the study cohort, we identified a subgroup of patients without chronic kidney disease (CKD), designated as the “No-CKD subgroup.” Patients were excluded if they had a baseline glomerular filtration rate (eGFR) below 60 mL/min/1.73 m², or a baseline diagnosis code for CKD or end-stage renal disease (ESRD).

A 7-day “washout” period was enforced following the index date to avoid misclassifying recurrent hyperkalemia. Consequently, follow-up commenced on the eighth day post-index and lasted for 12 months, divided into 12 consecutive 30-day analysis intervals (i.e., 0–1, 0–2, 0–3,.., 0–12 months). Patients were included in each interval up to, but not including the interval where the first censoring event occurred. Censoring events included the following: 1) the end of the study period on June 30, 2023; 2) the patient's death, either as an in-hospital event, recorded by a physician, or a change in vital status to “died”; or 3) the initiation of outpatient potassium binder therapy.

The following outcomes were described during the 12-month follow-up period: (1) the proportion of patients with a hyperkalemia recurrence, defined as serum K+ concentration > 5.0; (2) the number of hyperkalemia recurrences—where each recurrence was separated by at least 7 days; (3) the time in days to each hyperkalemia recurrence; and (4) the serum K+ concentration at each recurrence. Analyses were restricted to patients who remained uncensored throughout the 12-month follow-up period and were conducted for both the study cohort and the No-CKD subgroup. Additional analyses also evaluated hyperkalemia recurrence defined as serum K+ ≥ 5.5 mmol/L.

Baseline patient characteristics were utilized to describe patients included in the study cohort and No-CKD subgroup, and were assessed as potential risk factors of hyperkalemia recurrence. These characteristics included demographics (age, sex, and race), laboratory data (index serum K+ concentration and eGFR), whether the index hyperkalemia episode occurred in the inpatient or outpatient setting, a history of hyperkalemia (defined by K+ > 5.0 mmol/L or hyperkalemia diagnosis code), comorbidities (e.g., CKD, ESRD, malignancy, and diabetes mellitus), outpatient pharmacotherapy (e.g., anticoagulants, insulin, and antihypertensives), inpatient hyperkalemia therapy (e.g., inpatient K+ Binder, IV insulin/glucose, and IV/inhaled albuterol), and all-cause healthcare resource utilization (e.g., inpatient admissions and ED visits).

Baseline characteristics were ascertained within 24 months pre-index, inclusive of the index date. However, to ensure a close temporal relationship with the index hyperkalemia episode, some patient characteristics were collected within 12 months pre-index (e.g., eGFR, inpatient admissions, ED visits, and pharmacotherapy). Comorbidities and pharmacotherapy were classified by one inpatient/outpatient diagnosis code or one prescribing record, respectively, and described as binary variables. When multiple values of a given baseline variable (e.g., eGFR) were available during the baseline period, the value closest to or on the index date was used. For multiple values recorded on the same day, the most extreme value was selected (e.g., in the case of serum K+ in the inpatient setting). When no data for a specific characteristic was documented (e.g., no ICD-10-CM code for a comorbidity), the patient was classified as not having the condition. Baseline characteristics with missing values (e.g., BMI, serum bicarbonate, and urine Albumin-to-Creatinine Ratio) are neither presented nor evaluated as potential risk factors of recurrent hyperkalemia.

Continuous baseline patient characteristics were summarized using the mean, standard deviation (SD), and median. For risk factor modeling, these variables were dichotomized [e.g., age was modeled as age > 70 years (yes/no)]. Binary baseline patient characteristics were described by the frequency and percentage. Categorical patient characteristics, such as Race and CKD Stage (using eGFR), are presented in categories with frequency and percentage. For risk factor modeling, these categorical variables were converted into binary variables [e.g., Black race (yes/no), White race (yes/no) and CKD Stage 4–5 (yes/no)].

Binary outcomes, such as hyperkalemia recurrence (yes/no), were described by the proportion and the Clopper-Pearson 95% confidence interval (95% CI). Continuous outcomes, like the number of hyperkalemia recurrences and time to each hyperkalemia recurrence, were described using the mean (SD) and median.

Three models were developed to identify significant independent risk factors for hyperkalemia recurrence. Two of the models assessed different definitions of hyperkalemia (i.e., K+ > 5.0 mmol/L and K+ ≥ 5.5 mmol/L). The third model evaluated hyperkalemia recurrence (K+ > 5.0 mmol/L) in the No-CKD subgroup. For each model, a nine-step procedure was implemented utilizing multivariable logistic regression followed by Monte Carlo simulation for validation. The dependent variable in the model was hyperkalemia recurrence. Independent variables included baseline patient characteristics, which were classified as binary variables to facilitate both model development and interpretation. Details of the risk factor modeling procedure are provided in the Supplementary Material Table S1.

## Results

**Figure 2:**
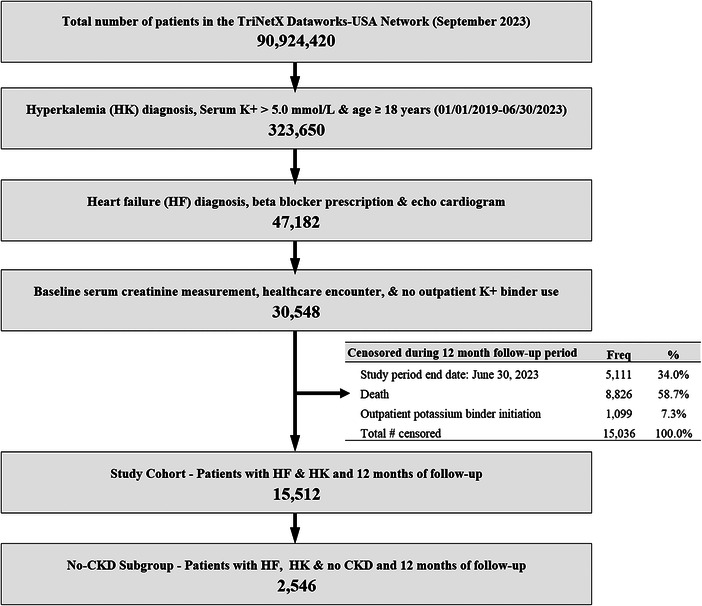
Study disposition and attrition.

The study cohort included 15,512 patients with HF and hyperkalemia who met the eligibility criteria and completed 12 months of follow-up (Figures 2, Illustration 1). The No-CKD subgroup, consisting of patients in the study cohort without CKD, included 2,546 individuals (16.4% of the study cohort). During the follow-up period, 15,036 patients were censored, with death being the primary reason (8,826 patients, 58.7% of censored cases). This high censoring rate due to mortality underscores the significant health risks in patients with heart failure and hyperkalemia.

**Illustration 1:**
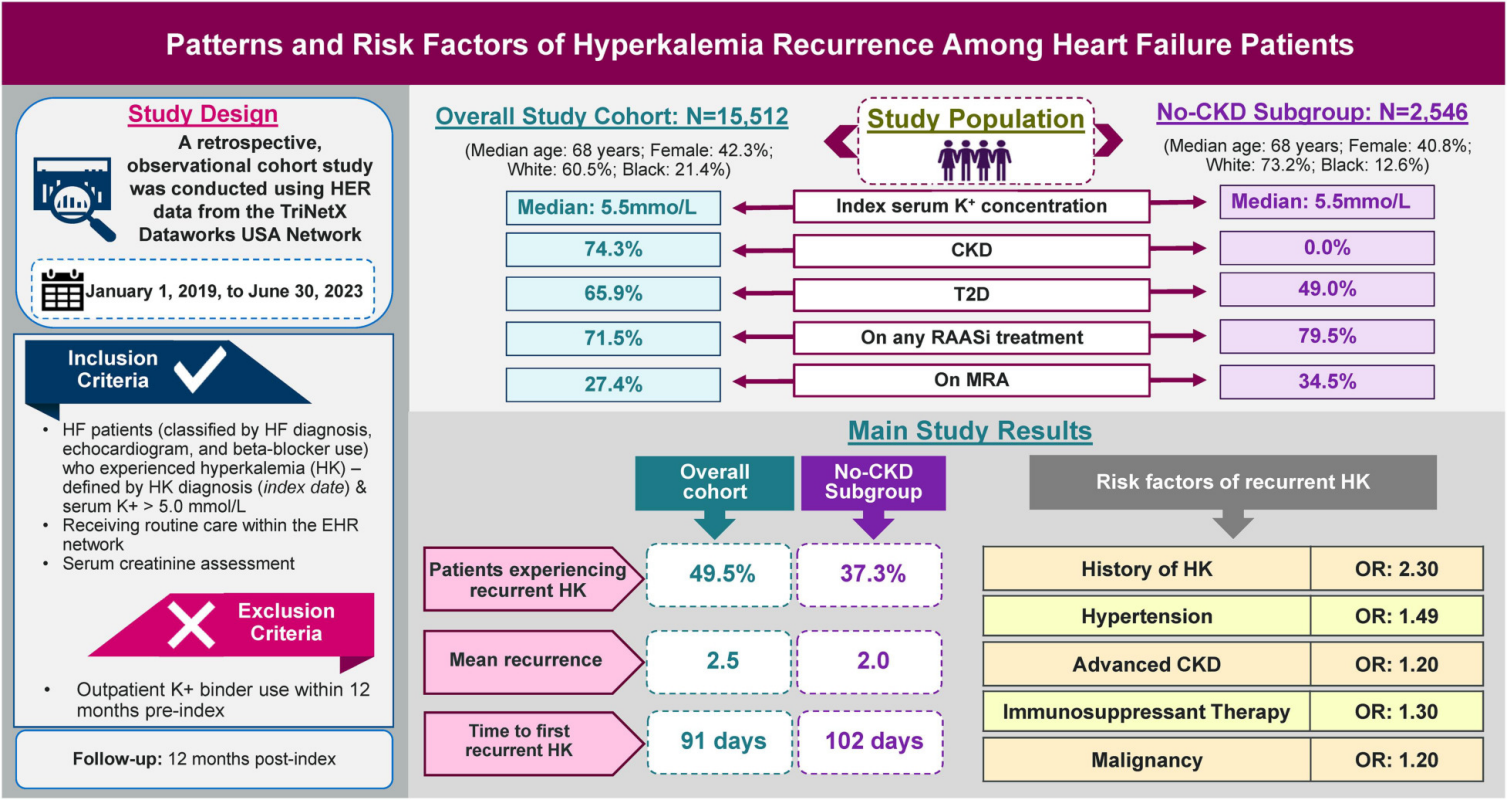
Patterns and risk factors of recurrent hyperkalemia among heart failure patients.

The study cohort had a median age of 68 years, with 42.3% female, and primarily consisted of White (60.5%) and Black (21.4%) patients (Table 1). The index hyperkalemia episode, predominantly identified during inpatient admission (68.6%), had a median serum K+ of 5.5 mmol/L, with 25.3% of cases classified as severe (K+ ≥ 6.0 mmol/L). Baseline comorbidities were highly prevalent, with a median Charlson Comorbidity Index of 10.0; most patients had a history of hyperkalemia (69.5%), CKD (74.3%, median eGFR 31.5 mL/min/1.73 m²), diabetes (65.9%), and hypertension (96.9%). During the 12 months pre-index, 82.9% of patients had at least one inpatient admission, and 27.3% used an inpatient K+ binder. Most patients (71.5%) had a prescribing record for a renin-angiotensin-aldosterone system inhibitor (RAASi). See Supplementary Material Table S2 for additional patient characteristics upon cohort entry (i.e., at the index date).

**Table 1:** Baseline patient characteristics—study cohort and No-CKD subgroup.

Patient characteristics	Study cohort	No-CKD subgroup
	*N* = 15,512	*N* = 2,546
Demographics	M (SD)M/*n*(%)	M (SD)M/*n*(%)
Age	66 (13) 68	67 (13) 68
Age 80 + years	2,690 (17.3%)	417 (16.4%)
Female	6,555 (42.3%)	1,038 (40.8%)
Race		
White	9,387 (60.5%)	1,863 (73.2%)
Black	3,314 (21.4%)	320 (12.6%)
Asian	568 (3.7%)	74 (2.9%)
Other/unknown	2,243 (14.5%)	289 (11.4%)
Index serum potassium (K+) concentration		
Index serum potassium (mmol/L)	5.7 (0.6) 5.5	5.6 (0.5) 5.5
Index serum potassium categories (mmol/L)		
K+ >5.0 to < 5.5	6,338 (40.9%)	1,241 (48.7%)
K+ 5.5 to < 6.0	5,255 (33.9%)	928 (36.4%)
K+ >=6.0	3,919 (25.3%)	377 (14.8%)
Inpatient index hyperkalemia episode	10,637 (68.6%)	1,337 (52.5%)
History of hyperkalemia	10,778 (69.5%)	1,375 (54.0%)
Estimated glomerular filtration rate (eGFR)		
MCQ eGFR	40 (31) 31	88 (17) 87
eGFR >90 (CKD Stage 1)	1,437 (9.3%)	1,077 (42.3%)
eGFR 60–89 (CKD Stage 2)	2,749 (17.7%)	1,469 (57.7%)
eGFR 45–59 (CKD Stage 3a)	1,681 (10.8%)	–
eGFR 30–44 (CKD Stage 3b)	2,132 (13.7%)	–
eGFR 15–29 (CKD Stage 4)	2,849 (18.4%)	–
eGFR <15 (CKD Stage 5)	4,664 (30.1%)	–
Comorbidities		
Charlson Comorbidity Index	10 (4) 10	7 (3) 7
Chronic kidney disease	11,521 (74.3%)	–
End Stage Renal Disease	4,771 (30.8%)	–
Hematologic or solid malignancy	3,759 (24.2%)	643 (25.3%)
Metastatic cancer	473 (3.0%)	96 (3.8%)
Chronic pulmonary disease	5,499 (35.4%)	939 (36.9%)
Diabetes Type 2	10,218 (65.9%)	1,248 (49.0%)
Serious cognitive impairment	1,483 (9.6%)	219 (8.6%)
Cerebrovascular disease	4,697 (30.3%)	641 (25.2%)
Peripheral vascular disease	6,100 (39.3%)	845 (33.2%)
Hypertension	15,032 (96.9%)	2,314 (90.9%)
Myocardial infarction	4,780 (30.8%)	655 (25.7%)
Liver disease	2,298 (14.8%)	368 (14.5%)
Peptic ulcer disease	1,149 (7.4%)	156 (6.1%)
Rheumatologic disease	1,382 (8.9%)	215 (8.4%)
Hemiplegia or paraplegia	832 (5.4%)	136 (5.3%)
Pharmacotherapy		
Inpatient/ED K+ Binder	4,236 (27.3%)	347 (13.6%)
Anticoagulant	6,323 (40.8%)	1,072 (42.1%)
Insulin	10,651 (68.7%)	1,309 (51.4%)
Non-insulin antidiabetic agent	4,311 (27.8%)	763 (30.0%)
SGLT2 inhibitor	909 (5.9%)	198 (7.8%)
Antiviral	730 (4.7%)	113 (4.4%)
Bronchodilator	9,602 (61.9%)	1,471 (57.8%)
Antihypertensive agent		
ACE inhibitor	6,095 (39.3%)	1,240 (48.7%)
ARB	5,296 (34.1%)	870 (34.2%)
ARNI	2,059 (13.3%)	443 (17.4%)
MRA	4,253 (27.4%)	878 (34.5%)
Any RAASi	11,097 (71.5%)	2,023 (79.5%)
Beta blocker	15,512 (100.0%)	2,546 (100.0%)
Loop diuretic	12,240 (78.9%)	1,893 (74.4%)
Thiazide	3,499 (22.6%)	398 (15.6%)
Immunosuppressant or immunomodulator	2,661 (17.2%)	343 (13.5%)
Lipid lowering agent	11,874 (76.5%)	1,848 (72.6%)
Sodium bicarbonate	5,083 (32.8%)	438 (17.2%)
Steroid	11,332 (73.1%)	1,746 (68.6%)
Inpatient hyperkalemia therapy		
IV calcium gluconate	3,638 (23.5%)	304 (11.9%)
IV insulin/glucose	7,651 (49.3%)	895 (35.2%)
IV/inhaled albuterol	6,671 (43.0%)	930 (36.5%)
IV sodium bicarbonate	2,013 (13.0%)	180 (7.1%)
IV loop diuretic	6,485 (41.8%)	963 (37.8%)
Inpatient dialysis	2,361 (15.2%)	11 (0.4%)
Healthcare resource utilization		
Inpatient admission	12,855 (82.9%)	1,895 (74.4%)
Emergency department visit	7,928 (51.1%)	1,139 (44.7%)
Ambulatory visit	14,797 (95.4%)	2,435 (95.6%)

CKD, chronic kidney disease; ED, emergency department; eGFR, estimated glomerular filtration rate; IV, intravenous; K+, serum potassium concentration; M(SD)M, mean, standard deviation, median; MCQ, mayo clinic quadratic (equation for estimating glomerular filtration rate); MRAs, mineralocorticoid receptor antagonists; Pre-Index, prior to the index hyperkalemia episode; RAASi, renin-angiotensin-aldosterone system inhibitor; SGLT2 Inhibitor, sodium-glucose cotransporter-2 inhibitor.

All values are mean (standard deviation) and median or frequency and percentage.

Table 1 shows the No-CKD subgroup had similar demographics (median age of 68 years and 41% female), but included fewer Black patients (12.6%). The median serum K+ was identical (5.5 mmol/L); however, fewer patients had severe hyperkalemia, (14.8%) or a history of hyperkalemia (54%). As expected, the median eGFR in the No-CKD subgroup was higher (86.6 mL/min/1.73 m²), reflecting preserved renal function. They also had a lower overall comorbidity burden, with a Charlson Comorbidity Index of 7.0, and a lower prevalence of diabetes (49.0%) and hypertension (90.0%).

Patterns of hyperkalemia recurrence are included in Table 2A (study cohort) and Table 2B (No-CKD subgroup). During the 12-month follow-up period, 49.5% of patients in the study cohort experienced at least one hyperkalemia recurrence (K+ > 5.0 mmol/L) and 28.4% had recurrent hyperkalemia defined as K+ ≥ 5.5 mmol/L, with an average of 2.5 recurrences per patient. The time to each successive recurrence decreased from 91 days for the first to 49 days for the fourth recurrence, and the proportion of patients experiencing each subsequent recurrence increased from 49.5% to 62.0%. The mean serum K+ concentration at each recurrence was ∼5.5 mmol/L. Additional results of hyperkalemia recurrence at each cumulative monthly interval are included in Supplementary Material Tables S3.

**Table 2:** Patterns of hyperkalemia recurrence: A. study cohort and B. No-CKD subgroup.

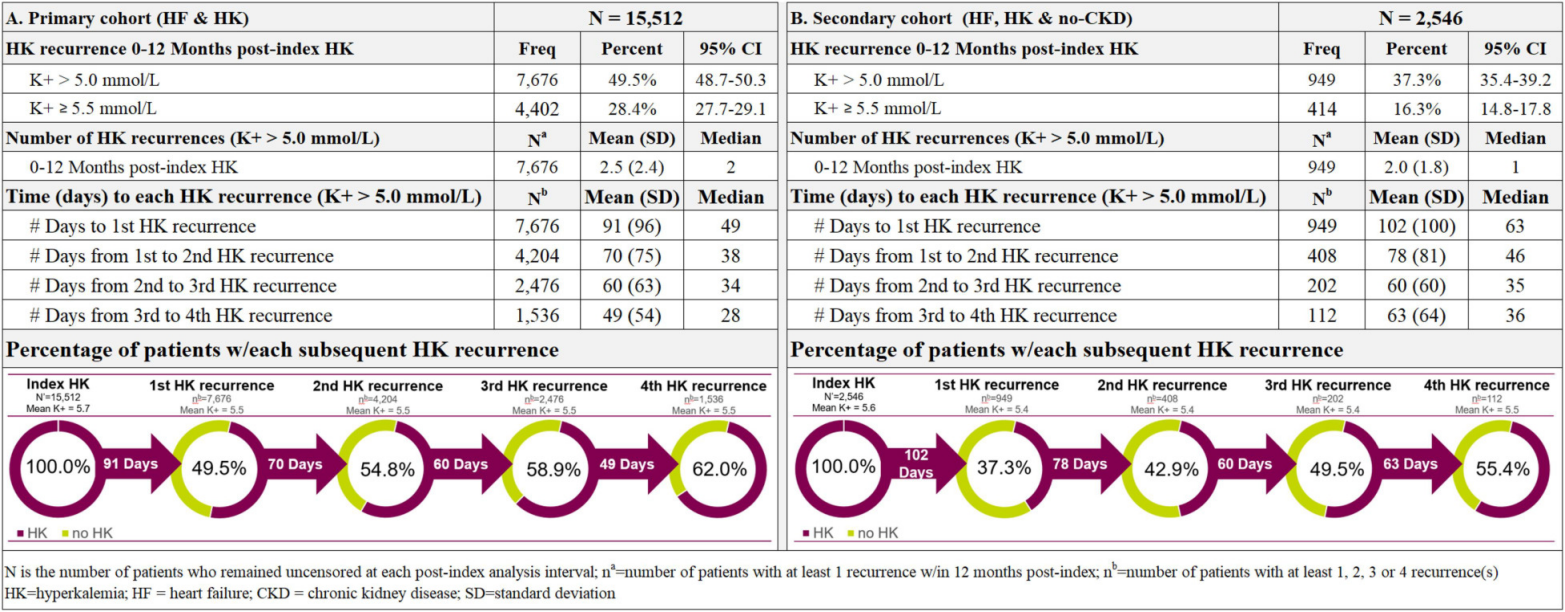

For the No-CKD subgroup (Table 2), 37.3% experienced recurrent hyperkalemia (K+ > 5.0 mmol/L), and 16.3% with K+ ≥ 5.5 mmol/L, averaging 2.0 recurrences. The time to each subsequent hyperkalemia recurrence decreased from 102 days for the first to 63 days for the fourth, with the proportion of patients with each subsequent recurrence increasing from 37.3% to 55.4%. Similarly, the mean serum K+ concentration at each hyperkalemia recurrence was ∼5.4 mmol/L.

**Figure 3:**
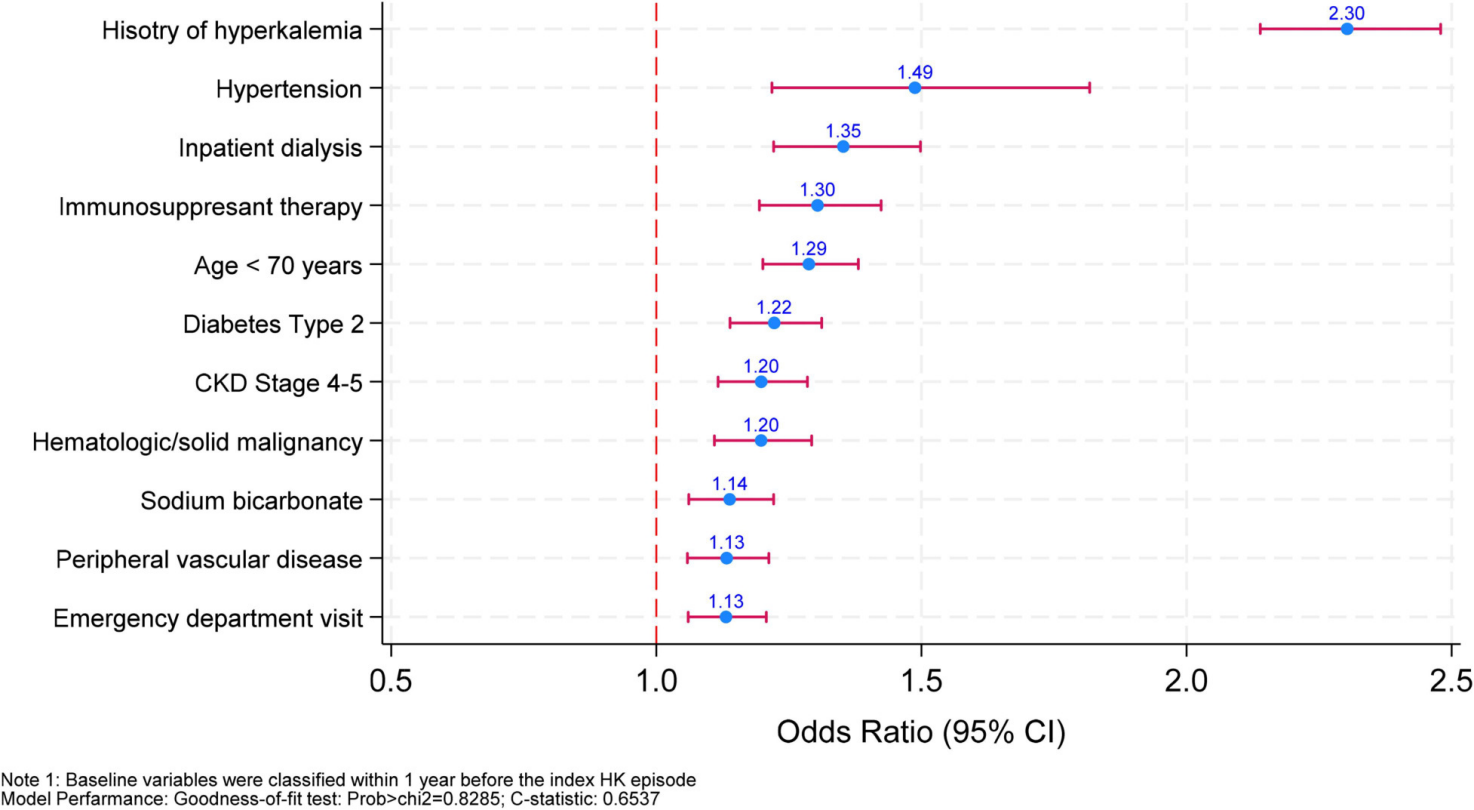
Risk factors of recurrent hyperkalemia (K+ > 5.0 mmol/L) Among heart failure patients (study cohort).

**Figure 4:**
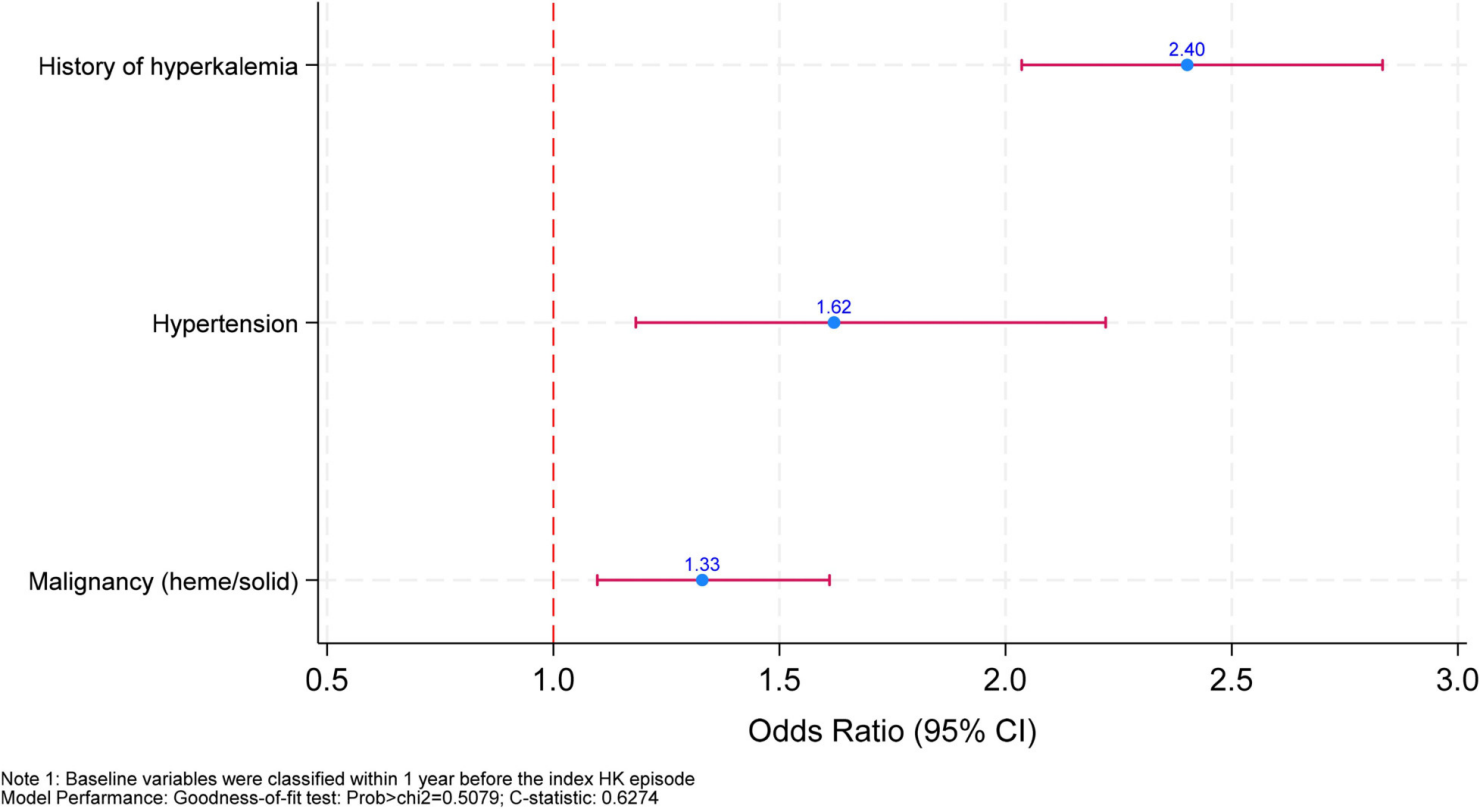
Risk factors of recurrent hyperkalemia (K+ > 5.0 mmol/L) Among heart failure patients without CKD (No-CKD subgroup).

Significant, independent risk factors for experiencing recurrent hyperkalemia (K+ > 5.0 mmol/L) in the study cohort are presented in Figure 3. Patients with a history of hyperkalemia were more than twice as likely to have recurrent hyperkalemia compared to those without a history of hyperkalemia, accounting for other risk factors in the model [Odds Ratio (95% CI): 2.3 (2.1–2.5)]. Patients with hypertension were 49% more likely to experience recurrent hyperkalemia compared to those without hypertension, while patients with CKD Stage 4–5 were 20% more likely to experience recurrent hyperkalemia compared to those with CKD Stage 1–3. Other risk factors included malignancy, treatment with an immunosuppressant agent, age < 70 years, and receiving inpatient dialysis. When recurrent hyperkalemia was defined as K+ ≥ 5.5 mmol/L (Supplementary Material Figure S1), additional notable risk factors in the study cohort, not previously identified, included Black race, inpatient admission, and severe hyperkalemia during the index hyperkalemia episode (K+ ≥ 6.0 mmol/L). For the No-CKD subgroup, risk factors for recurrent hyperkalemia (K+ > 5.0 mmol/L) included a history of hyperkalemia, hypertension, and malignancy (Figure 4). Additional results for each risk factor model, including univariate associations and Monte Carlo simulation findings, are provided in Supplementary Material Tables S4–S6.

Performance diagnostics to identify significant, independent risk factors of hyperkalemia recurrence among HF patients demonstrated moderate discrimination and acceptable calibration for all models. For hyperkalemia recurrence (K+ > 5.0 mmol/L), the models achieved a c-statistic of 0.65 (study cohort; Figure 3) and 0.63 (No-CKD subgroup; Figure 4), indicating a moderate ability to discriminate between patients who experienced hyperkalemia recurrence and those who did not. The Hosmer-Lemeshow goodness-of-fit test yielded values of 0.83 (study cohort) and 0.51 (No-CKD subgroup), suggesting that the model's predictions were well-calibrated. Together, these diagnostics reflect reasonable performance in accurately identifying risk factors of hyperkalemia recurrence among patients with HF and hyperkalemia.

## Discussion

Among patients with HF and hyperkalemia, this study offers comprehensive insights into the patterns and risk factors of recurrent hyperkalemia in those not treated with outpatient K+ binder therapy. The findings underscore the recurrent and potentially chronic nature of clinically important hyperkalemia in this population, with nearly half (49.5%) of patients experiencing at least one hyperkalemia recurrence (mean K+ > 5.0 mmol/L) within a year and a median of two recurrences. An accelerating pattern of hyperkalemia recurrence was observed, along with a greater percentage of patients experiencing each subsequent recurrence. Key risk factors for recurrent hyperkalemia (K+ > 5.0 mmol/L) included a history of hyperkalemia, which more than doubled the risk, as well as hypertension and advanced CKD. In the No-CKD subgroup, more than one-third of patients (37.3%) experienced recurrent hyperkalemia, with a similar pattern of shorter time intervals between subsequent recurrences; notably, a history of hyperkalemia and hypertension remained significant risk factors in this subgroup.

Comparing the findings of the present study to previous research is challenging due to the limited published literature on recurrence patterns and risk factors of hyperkalemia among HF patients. A 2023 meta-analysis of randomized controlled trials, that included HF patients with hyperkalemia (treated and untreated with K+ binders), reported the overall hyperkalemia recurrence (K+ > 5.5 mmol/L) risk was 21.4% for patients untreated with K+ binders (17), which aligns with the observed recurrence risk in the present study (K+ ≥ 5.5 mmol/L = 28.4%). Regarding risk factors for hyperkalemia recurrence, Crespo-Leiro et al., in a study of HF patients from a Spanish HF registry, found that increasing age, male gender, diabetes mellitus, stroke, lower eGFR, and older age were associated with an increased risk of hyperkalemia (18). Differences in the hyperkalemia risk factors (from the Spanish HF registry) compared to those in the present study may be explained by differences in the population and clinical settings. In the Crespo-Leiro et al. study, the clinical management of HF patients was conducted within the context of a long-term HF patient registry, which included a structured patient monitoring program and potassium surveillance/management protocols. In contrast, HF patients in the present study received routine clinical care from healthcare providers in the US with non-systematic surveillance and absent structured management of potassium monitoring or therapeutic intervention guidelines.

The recurrence of hyperkalemia in patients with HF may be driven by several postulated biologic mechanisms. Impaired renal potassium excretion, exacerbated by CKD and diabetes mellitus, likely plays a central role, as reduced eGFR limits the kidneys’ ability to excrete excess potassium. Additionally, the use of RAASi agents, essential for HF management, may further disrupt potassium homeostasis by reducing aldosterone-mediated excretion, particularly in patients with advanced HF. The observed increased risk in younger patients (<70 years) suggests potential contributions from unique pathophysiological factors, such as heightened sympathetic activation or altered cellular potassium handling, possibly linked to lifestyle or genetic predispositions. These mechanisms, interacting with the progressive nature of HF, may contribute to the accelerating pattern of hyperkalemia recurrence observed in this population.

The current study offers several notable strengths that enhance its validity and clinical relevance. Its retrospective, observational design utilized a large, real-world dataset from the TriNetX Dataworks USA Network, encompassing 52 healthcare organizations and approximately 90 million patients, providing a robust representation of HF patients with hyperkalemia, thereby allowing the findings to be more generalized to real-world clinical care of this population compared to studies implementing strict hyperkalemia management protocols (e.g., HF registry studies). The inclusion of both incident and prevalent hyperkalemia cases enabled a comprehensive assessment of recurrence risk factors, notably identifying a history of hyperkalemia as a primary driver. Furthermore, the exclusion of patients who received outpatient K+ binder therapy and the implementation of a 7-day washout period minimized confounding from treatment effects and reduced misclassification of recurrent events, strengthening the validity of the findings.

Despite these strengths, this study, utilizing EHR data from an “open” healthcare network, faced limitations including the potential for missing healthcare encounters (e.g., missing serum K+ assessments). This limitation was addressed to some extent by restricting eligibility to patients who regularly received care within the network and leveraging data from large, multi-site health systems to increase the likelihood of comprehensive data capture. To mitigate selection bias, the study cohort included all HF patients with a hyperkalemia event (diagnosis preceded by serum K+ > 5.0); however, analyses were restricted to patients not censored within 12 months, excluding approximately half of eligible patients, primarily due to death (58.7% or 8,826 out of 15,036). Restriction of the primary analyses to patients who completed 12 months of uncensored follow-up conditions on survival and non-initiation of outpatient potassium binders. Because death was the dominant reason for censoring, the observed recurrence patterns and risk associations may underestimate the burden of hyperkalemia in the broader population of heart failure patients who experience hyperkalemia. Future studies that retain all eligible patients and treat death as a competing risk would usefully complement the present estimates. Misclassification was mitigated by defining HF and hyperkalemia through a combination of diagnosis codes, procedure data (i.e., echocardiogram), pharmacotherapy (i.e., beta blocker use), and laboratory results, while excluding patients who received outpatient K+ binder therapy and censoring follow-up if such treatment was initiated, with a 7-day wash-out period enforced to differentiate recurrent hyperkalemia events. Although the 12-month follow-up period captured short- to medium-term recurrence patterns, longer-term follow-up may be warranted to better understand the full trajectory of hyperkalemia recurrence and its implications for patient outcomes in HF. Additionally, we modeled hyperkalemia recurrence as a binary outcome (any vs. none) within 12 months. Although this approach simplifies interpretation and facilitated validation, it does not exploit the timing or multiplicity of events. Analyses that treat hyperkalemia as a recurrent event process or that estimate rates while accounting for the timing of recurrences and the competing risk of death would provide a more complete description of the phenomenon. Finally, although dichotomization of continuous covariates can entail some loss of statistical precision and information, the resulting binary predictors enhance interpretability and support more direct translation into actionable clinical decision making. In future work, modeling strategies that retain continuous scales for key variables, together with multiple imputation procedures, merit careful consideration.

In conclusion, this study provides a comprehensive analysis of patients with HF and hyperkalemia, elucidating key patterns and significant risk factors for hyperkalemia recurrence. Notable findings reveal that nearly half of the patients experienced at least one hyperkalemia recurrence within a year, with an average serum K+ level of 5.5 mmol/L at each recurrence, indicating potentially chronic and clinically important hyperkalemia for some HF patients. Key risk factors for hyperkalemia recurrence encompass demographic, clinical, and therapeutic domains, with a history of hyperkalemia identified as the primary driver, alongside hypertension and advanced CKD. These findings emphasize the need for vigilant monitoring and proactive management strategies, particularly for high-risk patients, to mitigate potential adverse outcomes associated with hyperkalemia in HF.

## Data Availability

The datasets presented in this article are not readily available because the TriNetX Federated Data Network offers a subscription-based clinical data platform comprising worldwide de-identified electronic medical records that are accessible for querying. Detailed insights regarding the TriNetX Network are available at: https://www.ncbi.nlm.nih.gov/pmc/articles/PMC6816049/. For data requests, please contact join@trinetx.com, and access the network via https://live.trinetx.com/. Requests to access the datasets should be directed to join@trinetx.com.
