# Supplementary material for: Patterns and risk factors of hyperkalemia recurrence among heart failure patients
Source: Front Cardiovasc Med. 2026 Jun 11;13:1745946. doi: 10.3389/fcvm.2026.1745946 (PMC13296537; doi:10.3389/fcvm.2026.1745946)
Supplement: Supplementary file 1 [file Datasheet1.docx]

**Supplemental material**

**Manuscript title**

Patterns and Risk Factors of Hyperkalemia Recurrence Among Heart Failure Patients

**Supplemental material* table legend**

**Table S1.** Risk Factor Modeling Procedure

**Table S2.** Baseline Patient Characteristics at Index Date – Study Cohort & No-CKD Subgroup

**Table S3.** Hyperkalemia recurrence at each cumulative monthly interval: A. K+ > 5.0; B. K+ ≥ 5.5 mmol/L

**Table S4.** Results from Univariate Analyses and Monte Carlo Simulations: Study Cohort; K+ > 5.0 mmol/L

**Table S5.** Results from Univariate Analyses and Monte Carlo Simulations: No-CKD Subgroup; K+ > 5.0 mmol/L

**Table S6.** Results from Univariate Analyses and Monte Carlo Simulations: Study Cohort; K+ ≥ 5.5 mmol/L

**Table S7.** TRIPOD Checklist

**Supplemental material* Figure legend**

**Figure S1.** Risk Factors of Recurrent Hyperkalemia (K+ ≥ 5.5 mmol/L) Among Heart Failure Patients (Study Cohort)

**All supplemental material are intended for publication as an online data supplement*

**Table S1. Risk Factor Modeling Procedure**

| The nine-step procedure was implemented sequentially to identify significant, independent risk factors of hyperkalemia recurrence . The same procedure was used for the primary and secondary cohort. Cohort members who were uncensored for the entire follow-up duration (i.e., 0-12 month analysis interval) were included in the analysis set for the primary and secondary risk factor models. The “dependent variable” in the risk factor modeling procedure was hyperkalemia recurrence. The independent variables were baseline characteristics presented in **Table 1**. Every effort was made to dichotomize continuous and categorical variables. Using binary variables facilitated both the model building process and results interpretation. |
| --- |
| **1.**  Assess the missingness of all continuous baseline variables. baseline variables with any missingness were discarded and thereby excluded from further evaluation. |
| **2.**  Dichotomize continuous variables at a clinically relevant binary threshold (preferred method) or at the 75^th^ percentile of the continuous distribution. |
| **3.** Evaluate the prevalence of each binary baseline variable with the dependent variable. Binary baseline variables were excluded if any “cell count” of the 2x2 table was less than 10. For categorical variables, categories with cell counts less than 10 were “collapsed”. |
| **4.**  Using logistic regression, we estimated the univariate association for all baseline variables with the dependent variable. Baseline variables were ranked by the absolute value of the Wald statistic or “z-value”. |
| **5.**  For all significant baseline variables (abs[z-value] ≥ 1.96), we evaluated collinearity for each pairwise combination. When collinearity was identified (Pearson correlation coefficient > 0.8 absolute value), we retained the variable more significantly associated with the dependent variable based on the “z-value”. |
| **6.**  Starting with the most significant baseline variable (from the univariate analyses), we evaluated all subsequent baseline variables (in rank order based on abs[z-value]) for inclusion using the likelihood ratio test (LR test). This method used multivariable logistic regression to compare the fit of the nested model vs. the full model using the LR test. For each baseline variables, if the full model (including the baseline variable being evaluated) is a better fit (LR test p-value < 0.05) than the model excluding the baseline variable (i.e., the nested model), the baseline variable was retained. Otherwise, the baseline variable was dropped. This process was repeated for each baseline variable identified in step 5. |
| **7.**  Model validation was conducted using Monte Carlo (bootstrapping) simulation. Through 1,000 simulated datasets (using sampling with replacement), baseline variables were evaluated for inclusion in the validation model. Baseline variables “entered” and were “retained” in the validation model if the Wald statistic p-values were <0.05 and <0.1 for model entry and retention, respectively. The number of times each baseline variables was included (out of 1,000 simulations) was generated. |
| **8.** Baseline variables included in ≥ 80% of the bootstrap-generated models (from Step 7) were retained in the final predictive model. |
| **9.**  Performance diagnostics of the final predictive model were assessed. The final model’s ability to discriminate between patients with vs. without the hyperkalemia recurrence was assessed using the C-Statistic and area under the receiver operating characteristic (ROC) curve. Calibration (i.e., the agreement between observed and predicted risk) was assessed using the Hosmer-Lemeshow goodness-of-fit statistic (GOF). A small p-value (e.g., p<0.05) indicated the model was a poor fit. |

**Table S2. Baseline Patient Characteristics at Index Date – Study Cohort & No-CKD Subgroup**

| **Baseline Patient Characteristics** | **Study Cohort** | **No-CKD Subgroup** |
| --- | --- | --- |
|  | N = 30,548 | N = 4,282 |
| **Demographics** | **M(SD)M / n(%)** | **M(SD)M / n(%)** |
| Age | 68 (13) 69 | 67 (13) 69 |
| Age 80+ years | 6,052 (19.8%) | 760 (17.7%) |
| Female | 13,072 (42.8%) | 1,791 (41.8%) |
| Race: categorical |  |  |
| White | 18,941 (62.0%) | 3,126 (73.0%) |
| Black | 6,352 (20.8%) | 539 (12.6%) |
| Asian | 1,112 (3.6%) | 127 (3.0%) |
| Other/unknown | 4,143 (13.6%) | 490 (11.4%) |
| **Index serum potassium (K+) concentration** |  |  |
| Index serum potassium (mmol/L) | 5.7 (0.6) 5.6 | 5.6 (0.5) 5.5 |
| Index serum potassium categories (mmol/L) |  |  |
| K+ >5.0 to < 5.5 | 12,014 (39.3%) | 2,050 (47.9%) |
| K+ 5.5 to < 6.0 | 10,368 (33.9%) | 1,537 (35.9%) |
| K+ >=6.0 | 8,166 (26.7%) | 695 (16.2%) |
| Inpatient index hyperkalemia episode | 22,666 (74.2%) | 2,463 (57.5%) |
| History of hyperkalemia | 21,718 (71.1%) | 2,437 (56.9%) |
| **Estimated glomerular filtration rate (eGFR)** |  |  |
| MCQ eGFR: 12M Pre-Index | 38 (31) 28 | 88 (17) 86 |
| eGFR >90 (CKD Stage 1) | 2,448 (8.0%) | 1,789 (41.8%) |
| eGFR 60-89 (CKD Stage 2) | 4,876 (16.0%) | 2,493 (58.2%) |
| eGFR 45-59 (CKD Stage 3a) | 3,086 (10.1%) | - |
| eGFR 30-44 (CKD Stage 3b) | 4,153 (13.6%) | - |
| eGFR 15-29 (CKD Stage 4) | 6,077 (19.9%) | - |
| eGFR <15 (CKD Stage 5) | 9,908 (32.4%) | - |
| **Comorbidities** |  |  |
| Charlson Comorbidity Index | 10 (4) 10 | 8 (4) 7 |
| Chronic kidney disease | 23,423 (76.7%) | - |
| End Stage Renal Disease | 9,841 (32.2%) | - |
| Hematologic or solid malignancy | 8,602 (28.2%) | 1,220 (28.5%) |
| Metastatic cancer | 1,576 (5.2%) | 280 (6.5%) |
| Chronic pulmonary disease | 11,624 (38.1%) | 1,700 (39.7%) |
| Diabetes Type 2 | 20,250 (66.3%) | 2,067 (48.3%) |
| Serious cognitive impairment | 3,325 (10.9%) | 381 (8.9%) |
| Cerebrovascular disease | 9,937 (32.5%) | 1,148 (26.8%) |
| Peripheral vascular disease | 12,852 (42.1%) | 1,476 (34.5%) |
| Hypertension | 29,646 (97.0%) | 3,878 (90.6%) |
| Myocardial infarction | 10,189 (33.4%) | 1,154 (27.0%) |
| Liver disease | 5,158 (16.9%) | 668 (15.6%) |
| Peptic ulcer disease | 2,417 (7.9%) | 282 (6.6%) |
| Rheumatologic disease | 2,925 (9.6%) | 377 (8.8%) |
| Hemiplegia or paraplegia | 1,753 (5.7%) | 248 (5.8%) |
| **Pharmacotherapy w/in 12M Pre-Index** |  |  |
| Inpatient/ED K+ Binder | 9,389 (30.7%) | 680 (15.9%) |
| Anticoagulant | 13,154 (43.1%) | 1,858 (43.4%) |
| Insulin | 21,613 (70.8%) | 2,272 (53.1%) |
| Non-insulin antidiabetic agent | 8,750 (28.6%) | 1,329 (31.0%) |
| SGLT2 inhibitor | 2,556 (8.4%) | 484 (11.3%) |
| Antiviral | 1,839 (6.0%) | 267 (6.2%) |
| Bronchodilator | 19,757 (64.7%) | 2,579 (60.2%) |
| Antihypertensive agent |  |  |
| ACE inhibitor | 11,060 (36.2%) | 1,921 (44.9%) |
| ARB | 10,550 (34.5%) | 1,471 (34.4%) |
| ARNI | 4,338 (14.2%) | 764 (17.8%) |
| MRA | 8,199 (26.8%) | 1,459 (34.1%) |
| Any RAASi | 21,414 (70.1%) | 3,313 (77.4%) |
| Beta blocker | 30,548 (100.0%) | 4,282 (100.0%) |
| Loop diuretic | 24,522 (80.3%) | 3,261 (76.2%) |
| Thiazide | 7,258 (23.8%) | 705 (16.5%) |
| Immunosuppressant or immunomodulator | 5,621 (18.4%) | 650 (15.2%) |
| Lipid lowering agent | 23,584 (77.2%) | 3,094 (72.3%) |
| Sodium bicarbonate | 10,829 (35.4%) | 814 (19.0%) |
| Steroid | 23,059 (75.5%) | 3,066 (71.6%) |
| **Inpatient hyperkalemia therapy** |  |  |
| IV calcium gluconate | 7,751 (25.4%) | 611 (14.3%) |
| IV insulin/glucose | 16,080 (52.6%) | 1,629 (38.0%) |
| IV/inhaled albuterol | 13,846 (45.3%) | 1,672 (39.0%) |
| IV sodium bicarbonate | 4,450 (14.6%) | 337 (7.9%) |
| IV loop diuretic | 13,203 (43.2%) | 1,700 (39.7%) |
| Inpatient dialysis | 5,059 (16.6%) | 29 (0.7%) |
| **Healthcare resource utilization - "all cause"** |  |  |
| Inpatient admission | 26,057 (85.3%) | 3,290 (76.8%) |
| Emergency department visit | 15,815 (51.8%) | 1,975 (46.1%) |
| Ambulatory visit | 29,310 (95.9%) | 4,095 (95.6%) |
| CKD: Chronic Kidney Disease; ED: Emergency Department; eGFR: Estimated Glomerular Filtration Rate; IV: Intravenous; K+: Serum Potassium Concentration; M(SD)M: Mean, Standard Deviation, Median; MCQ: Mayo Clinic Quadratic (equation for estimating glomerular filtration rate); MRAs: Mineralocorticoid Receptor Antagonists; Pre-Index: Prior to the Index Hyperkalemia Episode; RAASi: Renin-Angiotensin-Aldosterone System Inhibitor; SGLT2 Inhibitor: Sodium-Glucose Cotransporter-2 Inhibitor. | | |

**Table S3. Hyperkalemia Recurrence at Each Cumulative Monthly Interval: A. K+ > 5.0; B. K+ ≥ 5.5 mmol/L**

| **A. HK recurrence (serum K+ > 5.0 mmol/L)** | | | | | |
| --- | --- | --- | --- | --- | --- |
| **Cohort at Index HK Episode** | **N = 30,548** |  | |  | |
| **Analysis interval** | **N'** | **K+ > 5.0 mmol/L** | | **95% CI** | |
|  |  | **#** | **% (P1)** | **LCL** | **UCL** |
| 0-1 Month | 25,515 | 6,198 | 24.3 | 23.8 | 24.8 |
| 0-2 Months | 23,913 | 7,236 | 30.3 | 29.7 | 30.8 |
| 0-3 Months | 22,684 | 7,792 | 34.4 | 33.7 | 35.0 |
| 0-4 Months | 21,665 | 8,129 | 37.5 | 36.9 | 38.2 |
| 0-5 Months | 20,771 | 8,289 | 39.9 | 39.2 | 40.6 |
| 0-6 Months | 19,945 | 8,339 | 41.8 | 41.1 | 42.5 |
| 0-7 Months | 19,164 | 8,307 | 43.3 | 42.6 | 44.1 |
| 0-8 Months | 18,446 | 8,249 | 44.7 | 44.0 | 45.4 |
| 0-9 Months | 17,641 | 8,148 | 46.2 | 45.5 | 46.9 |
| 0-10 Months | 16,920 | 8,009 | 47.3 | 46.6 | 48.1 |
| 0-11 Months | 16,185 | 7,844 | 48.5 | 47.7 | 49.2 |
| **0-12 Months** | **15,512** | **7,676** | **49.5** | **48.7** | **50.3** |
|  |  |  |  |  |  |
| **B. HK recurrence (serum K+ ≥ 5.5 mmol/L)** | | | | | |
| **Analysis interval** | **N'** | **K+ ≥ 5.5 mmol/L** | | **95% CI** | |
|  |  | **#** | **% (P1)** | **LCL** | **UCL** |
| 0-1 Month | 25,515 | 3,125 | 12.2 | 11.8 | 12.7 |
| 0-2 Months | 23,913 | 3,783 | 15.8 | 15.4 | 16.3 |
| 0-3 Months | 22,684 | 4,154 | 18.3 | 17.8 | 18.8 |
| 0-4 Months | 21,665 | 4,379 | 20.2 | 19.7 | 20.8 |
| 0-5 Months | 20,771 | 4,516 | 21.7 | 21.2 | 22.3 |
| 0-6 Months | 19,945 | 4,588 | 23.0 | 22.4 | 23.6 |
| 0-7 Months | 19,164 | 4,629 | 24.2 | 23.6 | 24.8 |
| 0-8 Months | 18,446 | 4,632 | 25.1 | 24.5 | 25.7 |
| 0-9 Months | 17,641 | 4,607 | 26.1 | 25.5 | 26.8 |
| 0-10 Months | 16,920 | 4,563 | 27.0 | 26.3 | 27.6 |
| 0-11 Months | 16,185 | 4,480 | 27.7 | 27.0 | 28.4 |
| **0-12 Months** | **15,512** | **4,402** | **28.4** | **27.7** | **29.1** |
| Abbreviations: N=the number of patients in the cohort at the start of follow-up; N' is the number of patients who remained uncensored at the end of each analysis interval following dietary counseling; 95% CI=95% confidence interval; LCL=lower confidence limit; UCL=upper confidence limit; P1 is the percentage of observed patients who had HK recurrence. | | | | | |

**Table S4. Results from Univariate Analyses and Monte Carlo Simulations: Study Cohort; K+ > 5.0 mmol/L**

**Table S5. Results from Univariate Analyses and Monte Carlo Simulations: No-CKD Subgroup; K+ > 5.0 mmol/L**

**Table S6. Results from Univariate Analyses and Monte Carlo Simulations: Study Cohort; K+ ≥ 5.5 mmol/L**

**Table S7. TRIPOD Checklist**

| **Section/Topic** | **Item** | **Checklist Item** | **Section** |
| --- | --- | --- | --- |
| **Title and abstract** | | | |
| Title | 1 | Identify the study as developing and/or validating a multivariable prediction model, the target population, and the outcome to be predicted. | Title |
| Abstract | 2 | Provide a summary of objectives, study design, setting, participants, sample size, predictors, outcome, statistical analysis, results, and conclusions. | Abstract |
| **Introduction** | | | |
| Background and objectives | 3a | Explain the medical context (including whether diagnostic or prognostic) and rationale for developing or validating the multivariable prediction model, including references to existing models. | Introduction |
|  | 3b | Specify the objectives, including whether the study describes the development or validation of the model or both. | Introduction |
| **Methods** | | | |
| Source of data | 4a | Describe the study design or source of data (e.g., randomized trial, cohort, or registry data), separately for the development and validation data sets, if applicable. | Methods |
|  | 4b | Specify the key study dates, including start of accrual; end of accrual; and, if applicable, end of follow-up. | Methods |
| Participants | 5a | Specify key elements of the study setting (e.g., primary care, secondary care, general population) including number and location of centres. | Methods |
|  | 5b | Describe eligibility criteria for participants. | Methods |
|  | 5c | Give details of treatments received, if relevant. |  |
| Outcome | 6a | Clearly define the outcome that is predicted by the prediction model, including how and when assessed. | Methods |
|  | 6b | Report any actions to blind assessment of the outcome to be predicted. |  |
| Predictors | 7a | Clearly define all predictors used in developing or validating the multivariable prediction model, including how and when they were measured. | Methods |
|  | 7b | Report any actions to blind assessment of predictors for the outcome and other predictors. |  |
| Sample size | 8 | Explain how the study size was arrived at. | Methods |
| Missing data | 9 | Describe how missing data were handled (e.g., complete-case analysis, single imputation, multiple imputation) with details of any imputation method. | Methods |
| Statistical analysis methods | 10a | Describe how predictors were handled in the analyses. | Methods |
|  | 10b | Specify type of model, all model-building procedures (including any predictor selection), and method for internal validation. | Methods |
|  | 10d | Specify all measures used to assess model performance and, if relevant, to compare multiple models. | Methods |
| Risk groups | 11 | Provide details on how risk groups were created, if done. |  |
| **Results** | | | |
| Participants | 13a | Describe the flow of participants through the study, including the number of participants with and without the outcome and, if applicable, a summary of the follow-up time. A diagram may be helpful. | Figure 2 |
|  | 13b | Describe the characteristics of the participants (basic demographics, clinical features, available predictors), including the number of participants with missing data for predictors and outcome. | Table 1 |
| Model development | 14a | Specify the number of participants and outcome events in each analysis. | Table 2 |
|  | 14b | If done, report the unadjusted association between each candidate predictor and outcome. | Table S4, S5 |
| Model specification | 15a | Present the full prediction model to allow predictions for individuals (i.e., all regression coefficients, and model intercept or baseline survival at a given time point). | Figure 3 & 4 |
|  | 15b | Explain how to the use the prediction model. | Results |
| Model performance | 16 | Report performance measures (with CIs) for the prediction model. | Figure 3 & 4 |
| **Discussion** | | | |
| Limitations | 18 | Discuss any limitations of the study (such as nonrepresentative sample, few events per predictor, missing data). | Discussion |
| Interpretation | 19b | Give an overall interpretation of the results, considering objectives, limitations, and results from similar studies, and other relevant evidence. | Discussion |
| Implications | 20 | Discuss the potential clinical use of the model and implications for future research. | Discussion |
| **Other information** | | | |
| Supplementary information | 21 | Provide information about the availability of supplementary resources, such as study protocol, Web calculator, and data sets. |  |
| Funding | 22 | Give the source of funding and the role of the funders for the present study. |  |

**Figure S1 Risk Factors of Hyperkalemia Recurrence (K+ ≥ 5.5 mmol/L) Among Heart Failure Patients (Study Cohort)**
